# Correction to “Dual Targeted Therapy as a Treatment Strategy for Refractory Psoriatic Arthritis: Real‐World Effectiveness and Safety From a Prospective Cohort”

**DOI:** 10.1002/acr2.90165

**Published:** 2026-10-01

Ribeiro AL , Al‐Zahir Z , Carrizo Abarza V , et al. Dual targeted therapy as a treatment strategy for refractory psoriatic arthritis: real‐world effectiveness and safety from a prospective cohort. ACR Open Rheumatol. 2026;8(8):e90084. doi:10.1002/acr2.90084 PMC1345803042576419

In the originally published article, the values in Table 2 were derived from an earlier analysis that was not restricted to complete cases. As described in the Methods, effectiveness outcomes were to be summarized only for patients with recorded values at baseline and at both prespecified follow‐up intervals of 3 to 6 months and 6 to 12 months. As a result, the medians and interquartile ranges published in Table 2 were incorrect. Separately, the interquartile range published for the baseline Patient Global value, 6.0 (6.9–8.1), contained a typographical error in its lower bound, which as printed exceeds the median.

**Table 2:** Effectiveness outcomes of dual biologic and targeted synthetic disease‐modifying antirheumatic drug therapy with biologic disease‐modifying antirheumatic drug combined with targeted synthetic disease‐modifying antirheumatic drug at 3 to 6 months and 6 to 12 months*

Outcome measure	n	Baseline, median (IQR)	First follow‐up (3–6 months), median (IQR)	Second follow‐up (6–12 months), median (IQR)
TJC 68	10	6.0 (1.3–9.8)	4.5 (1.5–5.8)	1.0 (0.0–1.0)
SJC 66	10	0.5 (0.0–4.5)	0.5 (0.0–1.8)	0.0 (0.0–0.0)
DAPSA	7	20.0 (15.1–36.5)	17.2 (11.2–20.8)	10.2 (7.0–13.5)
PASI	19	2.4 (1.1–6.3)	0.8 (0.1–1.2)	0.0 (0.0–0.1)
CRP, mg/L	7	4.1 (2.1–8.5)	5.0 (3.7–6.2)	2.9 (1.4–6.4)
BSA, %	19	3.0 (0.8–5.5)	1.0 (0.1–1.5)	0.0 (0.0–0.1)
Patient Global (0–10)	8	6.5 (6.0–7.3)	6.0 (3.5–8.0)	5.0 (2.8–6.3)
Patient‐reported pain (0–10)	9	6.0 (5.0–7.0)	6.0 (3.0–6.0)	4.0 (2.0–6.0)
Patient skin VAS (0–10)	9	6.0 (2.0–7.0)	1.0 (0.0–2.0)	1.0 (0.0–1.0)

* Baseline was defined as the assessment closest to initiation of dual biologic and targeted synthetic disease‐modifying antirheumatic drug therapy, allowing a window of up to six months before treatment start. Analyses were restricted to complete cases, that is, to patients with a recorded value for that outcome at baseline and at both prespecified follow‐up intervals of 3 to 6 months and 6 to 12 months; n therefore differs across outcomes. Effectiveness outcomes are reported as median (interquartile range) because of skewed distributions and small numbers, and no formal inferential pre–post testing was performed.

BSA, body surface area; DAPSA, Disease Activity Index for Psoriatic Arthritis; IQR, interquartile range; PASI, Psoriasis Area and Severity Index; CRP, C‐reactive protein; SJC, swollen joint count; TJC, tender joint count; VAS, visual analog scale.

The summary statistics have been recalculated using complete cases. Physician Global Assessment and PGA‐skin have been omitted from the corrected table because complete‐case data at all three time points were available for too few patients to permit meaningful summary, and body surface area and the patient skin visual analog scale, which were not reported in the original table, have been added because complete‐case data were available for these measures. Table 2 should have read as follows:

In addition, in the Results, under the subheading “Treatment patterns and duration”, the text “By three to six months, TJC and SJC showed reductions, accompanied by decreases in DAPSA and PASI. Improvements in PROs were more apparent at later follow‐up.” was incorrect. This should have read: “By three to six months, TJC, DAPSA and PASI showed reductions, whereas SJC was unchanged and declined only by six to twelve months. Improvements in PROs were also more apparent at later follow‐up.”

These corrections do not affect the abstract, the baseline characteristics reported in Table 1, the safety analyses reported in Tables 3 and 4, Figure 1, or the conclusions of the article.

We apologize for this error.

